# Supplementary material for: Plasticity in visual cortex is disrupted in a mouse model of tauopathy
Source: Commun Biol. 2022 Jan 20;5:77. doi: 10.1038/s42003-022-03012-9 (PMC8776781; doi:10.1038/s42003-022-03012-9)
Supplement: Supplementary file 2 — Supplemental Information [file 42003_2022_3012_MOESM2_ESM.pdf]

Supplementary Material

		VEP Amplitude (μV)	Negative amplitude (μV)	Positive amplitude (μV)	Time to negative peak (ms)	Time to positive peak (ms)	FWHM negative peak (ms)	FWHM positive peak (ms)	Decay slope
Stationary	Tau- Mean±SEM	5m: 173±12<br>8m: 167±21	5m: -86±7<br>8m: -83±11	5m: 86±6<br>8m: 84±11	5m: 43±1<br>8m: 52±5	5m: 120±5<br>8m: 138±4	5m: 25±3<br>8m: 31±4	5m: 47±2<br>8m: 55±1	5m: -0.06±0.004<br>8m: -0.06±0.005
	Tau+ Mean±SEM	5m: 211±14<br>8m: 142±18	5m: -113±11<br>8m: -72±9	5m: 98±9<br>8m: 71±11	5m: 43±1<br>8m: 46±1	5m: 119±11<br>8m: 132±10	5m: 24±4<br>8m: 23±2	5m: 56±3<br>8m: 63±8	5m: -0.05±0.004<br>8m: -0.05±0.006
	Two-way ANOVA	Genotype:<br>F=0.16, p=0.69<br>Age:<br>F=4.7, p=0.03<br>Genotype*Age:<br>F=3.49, p=0.07	Genotype:<br>F=0.7, p=0.4<br>Age:<br>F=5.75, p=0.02<br>Genotype*Age:<br>F=4.3, p=0.04	Genotype:<br>F=0.01, p=0.9<br>Age:<br>F=2.28, p=0.1<br>Genotype*Age:<br>F=1,67, p=0.2	Genotype:<br>F=1.2, p=0.28<br>Age:<br>F=4.26, p=0.04<br>Genotype*Age:<br>F=0.89, p=0.35	Genotype:<br>F=0.19, p=0.67<br>Age:<br>F=3.96, p=0.05<br>Genotype*Age:<br>F=0.07, p=0.79	Genotype:<br>F=1.64, p=0.2<br>Age:<br>F=0.39, p=0.53<br>Genotype*Age:<br>F=1.12, p=0.29	Genotype:<br>F=3.19, p=0.08<br>Age:<br>F=2.62, p=0.11<br>Genotype*Age:<br>F=0.02, p=0.88	Genotype:<br>F=3.4, p=0.07<br>Age:<br>F=0.01, p=0.94<br>Genotype*Age:<br>F=0.59, p=0.45
Running	Tau- Mean±SEM	5m: 138±7<br>8m: 107±11	5m: -78±7<br>8m: -68±10	5m: 60±3<br>8m: 39±6	5m: 55±9<br>8m: 53±3	5m: 118±5<br>8m: 135±17	5m: 42±4<br>8m: 39±6	5m: 45±4<br>8m: 58±10	5m: -0.08±0.02<br>8m: -0.02±0.005
	Tau+ Mean±SEM	5m: 114±13<br>8m: 104±13	5m: -71±10<br>8m: -58±7	5m: 43±5<br>8m: 46±8	5m: 46±6<br>8m: 48±3	5m: 115±11<br>8m: 113±9	5m: 19±4<br>8m: 27±4	5m: 44±7<br>8m: 43±6	5m: -0.06±0.01<br>8m: -0.06±0.02
	Two-way ANOVA	Genotype:<br>F=1.42, p=0.24<br>Age:<br>F=3.21, p=0.08<br>Genotype*Age:<br>F=0.78, p=0.38	Genotype:<br>F=1.03, p=0.31<br>Age:<br>F=1.75, p=0.19<br>Genotype*Age:<br>F=0.05, p=0.82	Genotype:<br>F=0.67, p=0.42<br>Age:<br>F=2.33, p=0.13<br>Genotype*Age:<br>F=4.04, p=0.05	Genotype:<br>F=1.57, p=0.22<br>Age:<br>F=0, p=0.99<br>Genotype*Age:<br>F=0.14, p=0.7	Genotype:<br>F=1.25, p=0.27<br>Age:<br>F=0.46, p=0.5<br>Genotype*Age:<br>F=0.68, p=0.41	Genotype:<br>F=15.59,<br>p=0.3*10-4<br>Age:<br>F=0.24, p=0.62<br>Genotype*Age:<br>F=1.58, p=0.21	Genotype:<br>F=1.27, p=0.26<br>Age:<br>F=0.76, p=0.39<br>Genotype*Age:<br>F=1.07, p=0.3	Genotype:<br>F=0.52, p=0.47<br>Age:<br>F=2.3, p=0.14<br>Genotype*Age:<br>F=4.07, p=0.05

Supplementary Table 1: VEP signal measures for Tau- and Tau+ animals on day 1. Average values (mean±sem) and statistics for 5-month old and 8-month old Tau- and Tau+ mice of the VEP amplitude, amplitude of the negative peak from the baseline, amplitude of the positive peak from the baseline, time to negative peak since stimulus onset, time to positive peak, full width at half maximum (FWHM) of the negative and positive peaks and decay slope after fitting an exponential to the values following the positive peak. Values are calculated during just stationary or just running epochs. Significance (p<=0.05) is indicated with red.

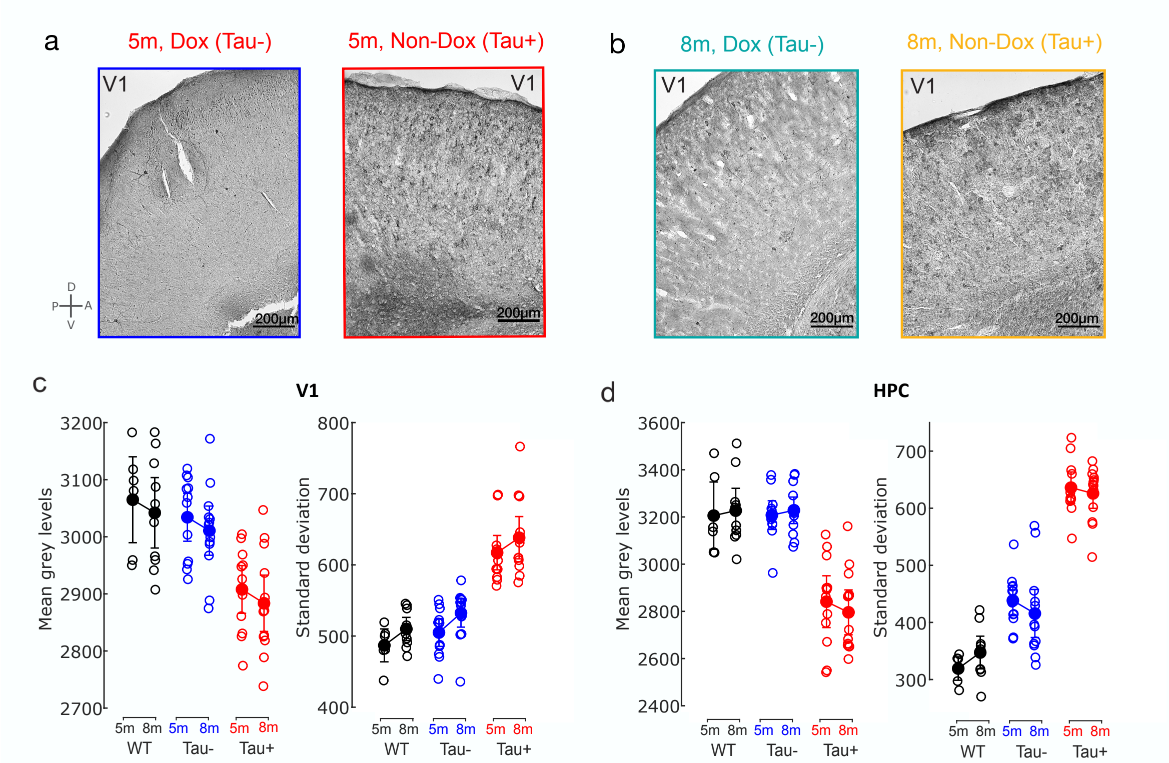

Supplementary Figure 1: Immunohistochemical profiling of 5- and 8-month old rTg4510 mice. a,b. Representative immunohistochemical images from the V1 of Tau- mice receiving doxycycline (Dox) treatment and Tau+ mice (Non-Dox) at 5 months old (**a**) and 8 months old (**b**). **c.** Average of the mean grey

levels (left) and standard deviation (right) of ROIs selected in V1 for each animal (Methods). Data are represented as mean±2*SEM. **d.** Same as in C. for hippocampal ROIs. Tau+ animals were characterised by a smaller mean and larger standard deviation compared to Tau- and WT animals, confirming that the doxycycline treatment was successful.

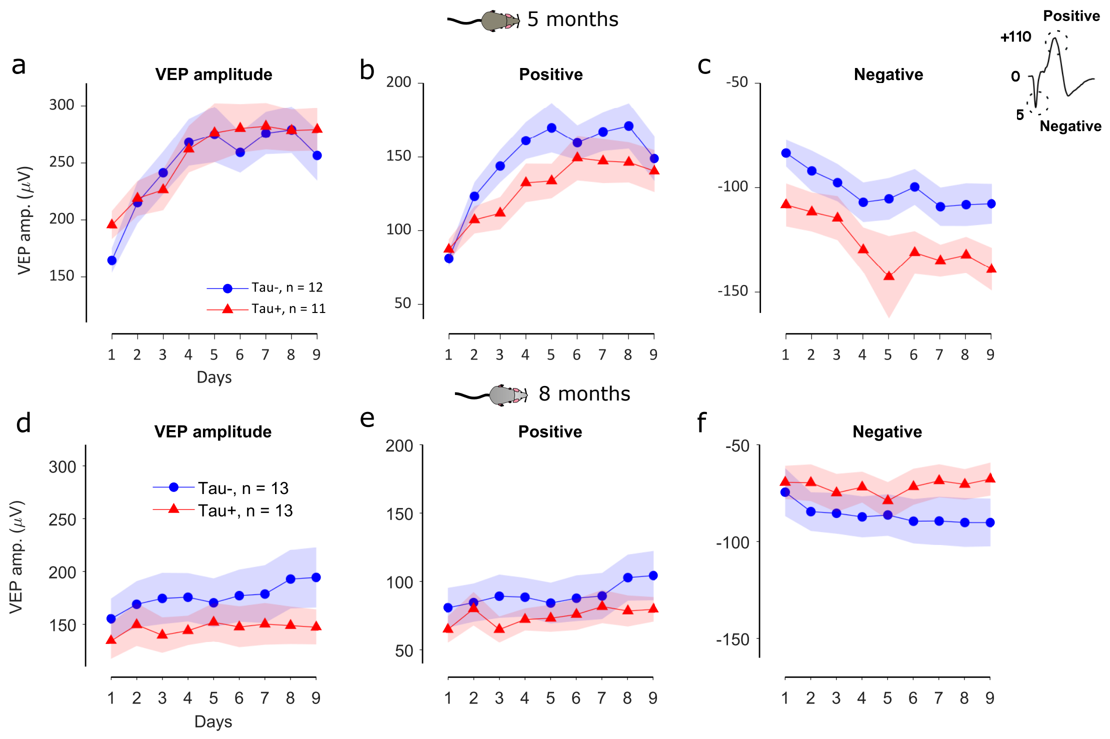

Supplementary Figure 2: Differences in visual plasticity between Tau+ and Tau- animals are attributed mainly to the positive deflection of the VEP signal. a. Average VEP amplitude, defined as the difference between the positive and negative peaks of the VEP signal, across days. **b.** Difference between the positive deflection of the VEP signal and the baseline as a function of days, averaged across Tau+ and Tau- animals. **c.** Difference between the negative deflection of the VEP signal and the baseline as a function of days, averaged across the groups of animals. **d-f.** Same for 8-month old animals. Shaded area represents the mean±SEM.

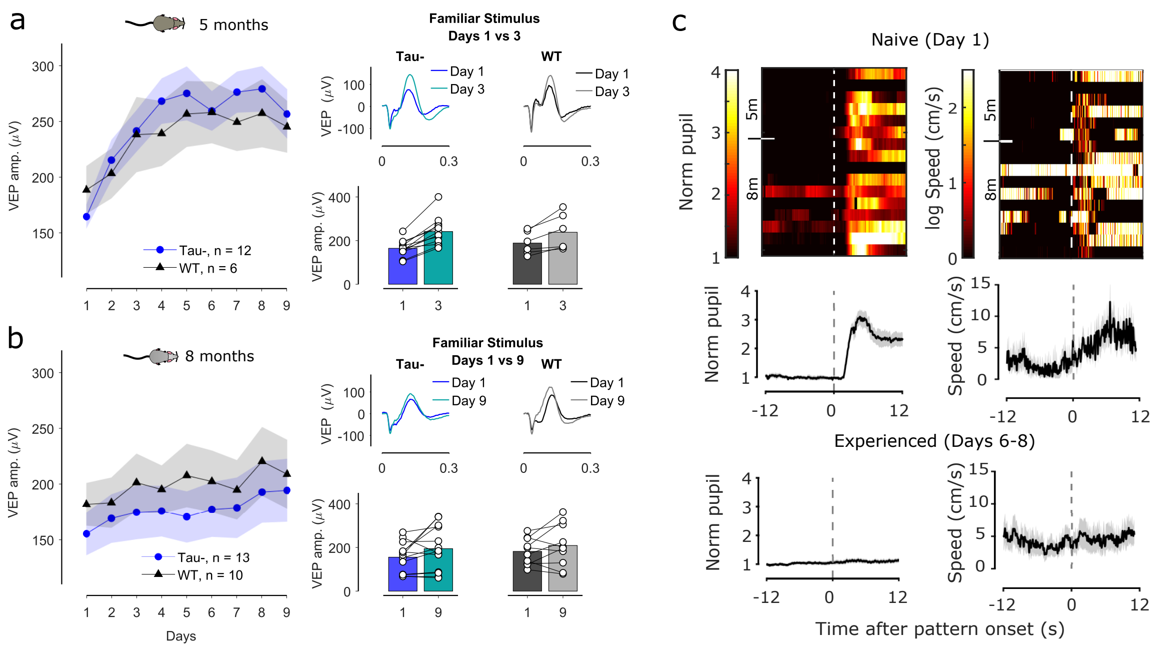

Supplementary Figure 3: VEP and behavioural responses are similar in Tau- and Wild Type (WT) mice. a. Average VEP amplitude as a function of days for 5-month old Tau- mice (blue) and WT littermates (black). The panels on the right show the average VEP signal on top and the VEP amplitude of individual animals on the bottom for days 1 and 3. VEPs significantly increased by day 3 relative to day 1 for the familiar stimulus for both Tau- and WT animals. **b.** Average VEP amplitude as a function of days for 8-month old Tau- mice (blue) and WT littermates (black). The panels on the right show the average VEP signal and the VEP amplitude of individual animals for days 1 and 9. WT mice had on average a larger VEP amplitude than Tau- mice but the VEPs increased at a similar rate as a function of days. The rate and magnitude of potentiation was reduced compared to 5-month old animals. **c.** Left: Images and average responses of the normalized pupil to the onset of the stimulus of the first block presented for naive (top) and experienced (bottom) WT mice. Right: Images and average responses of the movement speed. Shaded area represents the mean±SEM.

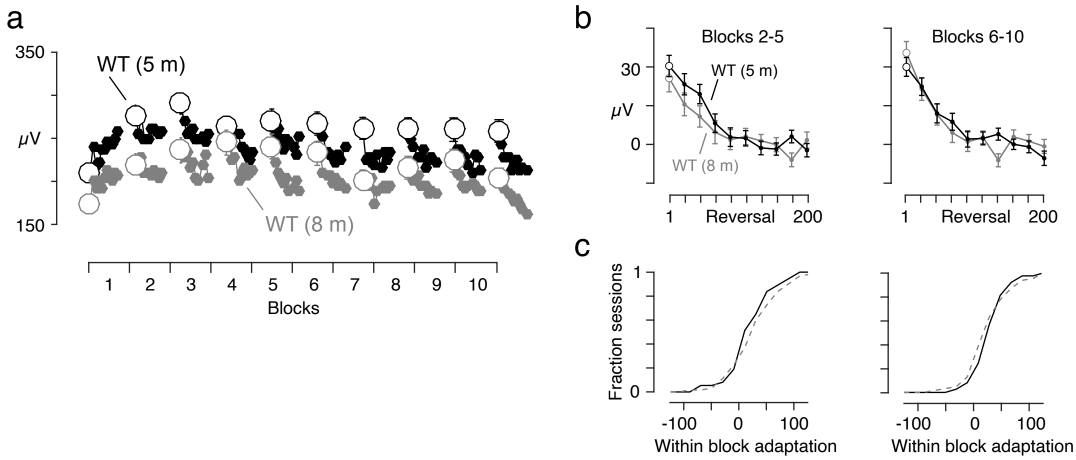

Supplementary Figure 4: Adaptation in WT animals does not reduce with age (related to Figure 4). a. Average VEP amplitude as a function of block number for 5-month old WT mice (black) and 8-month old WT mice (grey). The VEP amplitude was calculated from non-overlapping averages of 20 reversals. With the notable exception of the first block, VEPs showed a reduction of responses within each block, consistent with classic sensory adaptation effects. **b.** Average VEP responses for blocks 2-5 (Left) and blocks 6-10 (Right) on days 2-8. **c.** Cumulative histograms of the fitted amplitudes showing no significant differences between 5- and 8-month old WT mice for either early (2-5) or late (6-10) blocks.

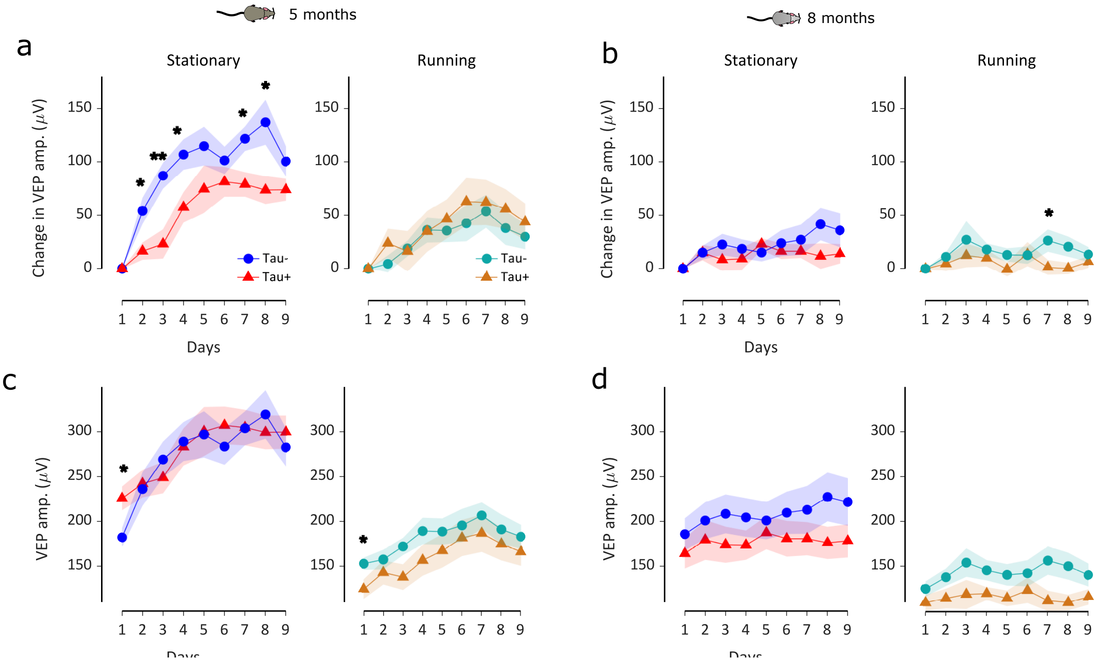

Supplementary Figure 5: Running reduces the VEP amplitude but does not affect stimulus-response potentiation in either Tau+ or Tau- animals. a. Difference in the VEP amplitude from day 1 for the familiar stimulus for Tau- (left) and Tau+ (right) 5-month old animals over the course of days calculated considering only stationary or running epochs. **b.** Same as in A for 8-month old animals. **c.** Unnormalized VEP amplitude as a function of days for 5-month old Tau- (left) and Tau+ (right) mice calculated considering only stationary or running epochs. **d.** Same as in C for 8-month old animals. Shaded areas represent the mean±SEM.

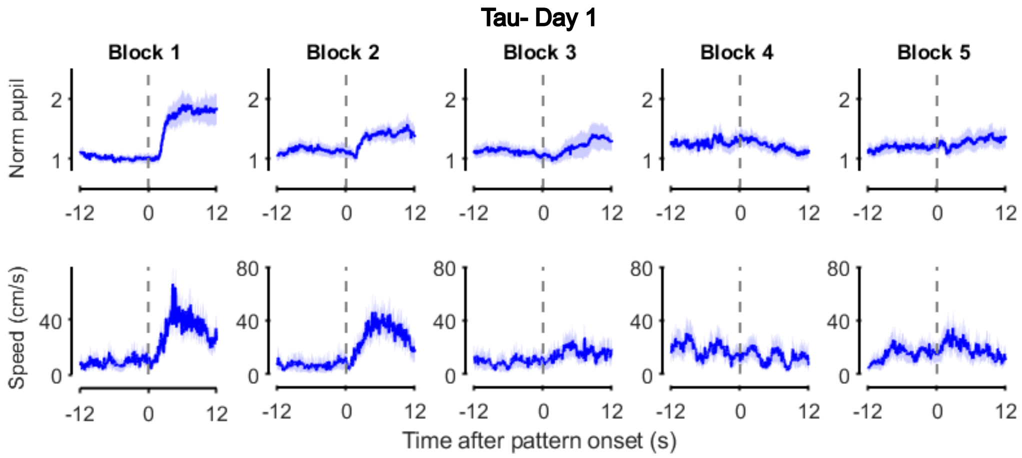

Supplementary Figure 6: Visual evoked behaviour habituates quickly in Tau- animals. Mean (±SEM) normalized pupil responses (top) and movement speed (bottom) to the onset of the stimulus during the first five blocks of presentation for naive Tau- animals (day 1).
