# Supplementary material for: Plasticity in visual cortex is disrupted in a mouse model of tauopathy
Source: Commun Biol. 2022 Jan 20;5:77. doi: 10.1038/s42003-022-03012-9 (PMC8776781; doi:10.1038/s42003-022-03012-9)
Supplement: Supplementary file 3 — Description of Additional Supplementary Files [file 42003_2022_3012_MOESM3_ESM.pdf]

Description of Additional Supplementary Files

File name: Supplementary Data 1
Description: Tau burden, brain weights and VEP traces for the familiar stimulus on Day 1 for each animal.

File name: Supplementary Data 2
Description: Change in VEP amplitude from Day 1, absolute VEP amplitudes on each day and VEP traces on days 1,3 and 9 for the familiar and unfamiliar stimuli for 5-month old Tau- and Tau+ animals.

File name: Supplementary Data 3
Description: Change in VEP amplitude from Day 1, absolute VEP amplitudes on each day and VEP traces on days 1 and 9 for the familiar and unfamiliar stimuli for 8-month old Tau- and Tau+ animals.

File name: Supplementary Data 4
Description: VEP amplitudes of each animal for each subblock on each day (10 subblocks/block, 10 blocks/day = 100 subblocks/day).

File name: Supplementary Data 5
Description: VEP traces for the familiar stimulus on day 1, VEP amplitudes on each day and change in the VEP amplitude from day 1 for Tau- and Tau+ 5-month-old animals during stationary and running epochs. Impact for each regressor (days, blocks, speed, pupil) for each animal.

File name: Supplementary Data 6
Description: Normalized pupil responses and speed responses to the onset of the stimulus of the first block for naïve (day 1) and experienced (days 6-8) Tau- and Tau+ animals.
