# Supplementary material for: Plasticity in visual cortex is disrupted in a mouse model of tauopathy
Source: Commun Biol. 2022 Jan 20;5:77. doi: 10.1038/s42003-022-03012-9 (PMC8776781; doi:10.1038/s42003-022-03012-9)
Supplement: Supplementary file 10 — Reporting Summary [file 42003_2022_3012_MOESM10_ESM.pdf]

nature research

Corresponding author(s): Amalia Papanikolaou

Last updated by author(s): Nov 19, 2021

Reporting Summary

Nature Research wishes to improve the reproducibility of the work that we publish. This form provides structure for consistency and transparency in reporting. For further information on Nature Research policies, see our Editorial Policies and the Editorial Policy Checklist.

Statistics

For all statistical analyses, confirm that the following items are present in the figure legend, table legend, main text, or Methods section.

n/a	Confirmed	
☐	☒	The exact sample size (*n*) for each experimental group/condition, given as a discrete number and unit of measurement
☐	☒	A statement on whether measurements were taken from distinct samples or whether the same sample was measured repeatedly
☐	☒	The statistical test(s) used AND whether they are one- or two-sided<br>*Only common tests should be described solely by name; describe more complex techniques in the Methods section.*
☐	☒	A description of all covariates tested
☐	☒	A description of any assumptions or corrections, such as tests of normality and adjustment for multiple comparisons
☐	☒	A full description of the statistical parameters including central tendency (e.g. means) or other basic estimates (e.g. regression coefficient) AND variation (e.g. standard deviation) or associated estimates of uncertainty (e.g. confidence intervals)
☐	☒	For null hypothesis testing, the test statistic (e.g. *F*, *t*, *r*) with confidence intervals, effect sizes, degrees of freedom and *P* value noted<br>*Give P values as exact values whenever suitable.*
☒	☐	For Bayesian analysis, information on the choice of priors and Markov chain Monte Carlo settings
☒	☐	For hierarchical and complex designs, identification of the appropriate level for tests and full reporting of outcomes
☒	☐	Estimates of effect sizes (e.g. Cohen's *d*, Pearson's *r*), indicating how they were calculated

Our web collection on statistics for biologists contains articles on many of the points above.

Software and code

Policy information about availability of computer code

Data collection	Visual stimuli were generated using open source package BonVision using open source software Bonsai. Open Ephys acquisition.
Data analysis	All data were analysed using custom software written in MATLAB (MathWorks)

For manuscripts utilizing custom algorithms or software that are central to the research but not yet described in published literature, software must be made available to editors and reviewers. We strongly encourage code deposition in a community repository (e.g. GitHub). See the Nature Research guidelines for submitting code & software for further information.

Data

Policy information about availability of data

All manuscripts must include a data availability statement. This statement should provide the following information, where applicable:

- Accession codes, unique identifiers, or web links for publicly available datasets
- A list of figures that have associated raw data
- A description of any restrictions on data availability

The datasets generated during and/or analysed during the current study are available from the corresponding author on reasonable request.

Field-specific reporting

Life sciences study design

All studies must disclose on these points even when the disclosure is negative.

Sample size	We did not know the variability of SRP / adaptation measures going into this study so we tried to assess the former from previous data: LFP measurements of SRP from 20 animals show response increase in LFP of ca. 249% (s.d. 82) over 5 or more days of induction, and we have obtained similar values in 2 preliminary experiments on C57BL6 mice. We would therefore require 10 animals in each of the DOX and No-DOX groups to detect a change of 40%.
Data exclusions	We have excluded the electrophysiological data from one Tau+ mouse for which histological analysis revealed that electrode was placed too deep in V1. We did not exclude any other data except in those cases where the recordings were catastrophically corrupted.
Replication	We obtained measurements from two separate batches of animals for each phenotype and each age. Results were similar between the two batches.
Randomization	We randomised animal selection that had the DOX diet by cage.
Blinding	Analysis of immunohistochemistry images was performed blinded to the animal's phenotype.

Reporting for specific materials, systems and methods

We require information from authors about some types of materials, experimental systems and methods used in many studies. Here, indicate whether each material, system or method listed is relevant to your study. If you are not sure if a list item applies to your research, read the appropriate section before selecting a response.

Materials & experimental systems

n/a	Involved in the study	
☐	☒	Antibodies
☒	☐	Eukaryotic cell lines
☒	☐	Palaeontology and archaeology
☐	☒	Animals and other organisms
☒	☐	Human research participants
☒	☐	Clinical data
☒	☐	Dual use research of concern

Methods

n/a	Involved in the study	
☒	☐	ChIP-seq
☒	☐	Flow cytometry
☒	☐	MRI-based neuroimaging

Antibodies

Antibodies used	A primary antibody for tau phosphorylated at serine 202 (mouse monoclonal AT8, 1:1000, Thermo Fisher Scientific) was used for immonohistochemistry.
Validation	This Antibody was verified by the supplier by Cell treatment to ensure that the antibody binds to the antigen stated.

Animals and other organisms

Policy information about studies involving animals; ARRIVE guidelines recommended for reporting animal research

Laboratory animals	rTg4510 transgenic mice and Wild Type littermates, males, 22-26 weeks, 31-35 weeks
Wild animals	n/a
Field-collected samples	n/a
Ethics oversight	All experiments were performed in accordance with the Animals (Scientific Procedures) Act 1986 (United Kingdom) and Home Office (United Kingdom) approved project and personal licenses. The experiments were approved by the University College London Animal Welfare Ethical Review Board under Project License 70/8637.

Note that full information on the approval of the study protocol must also be provided in the manuscript.
